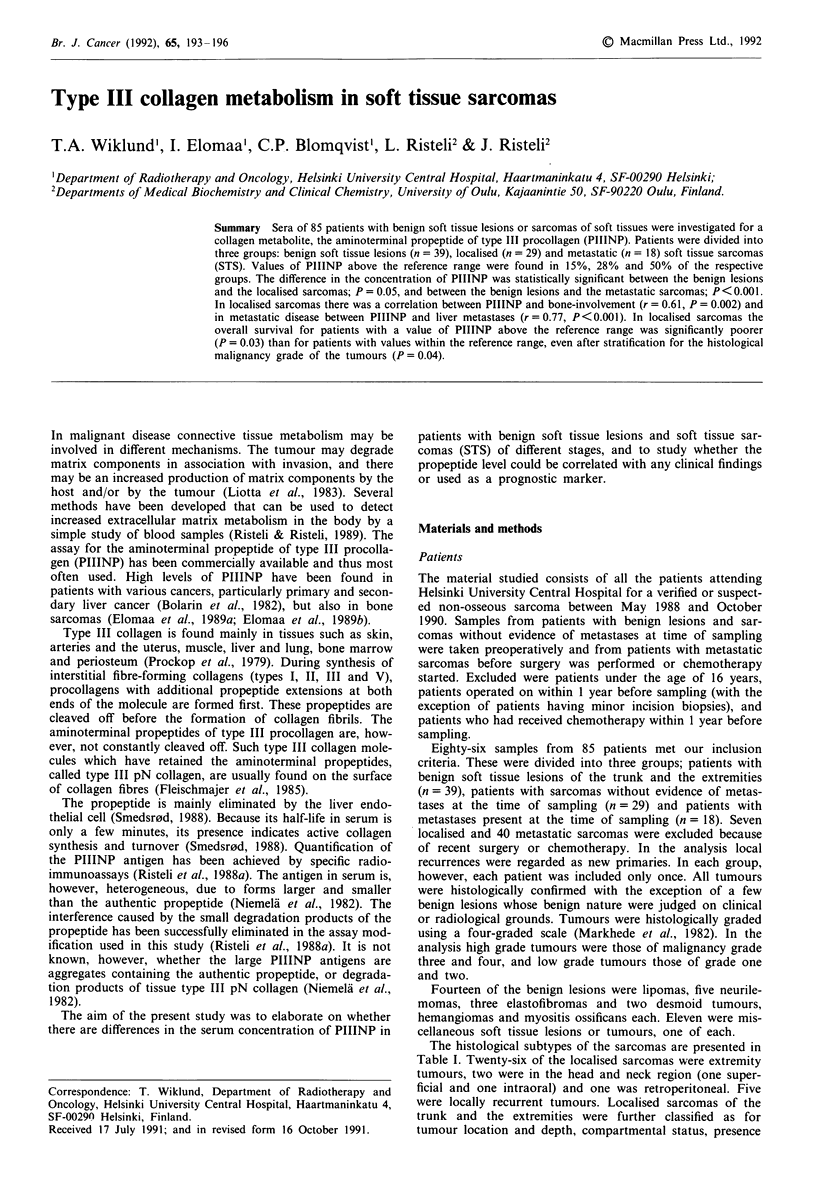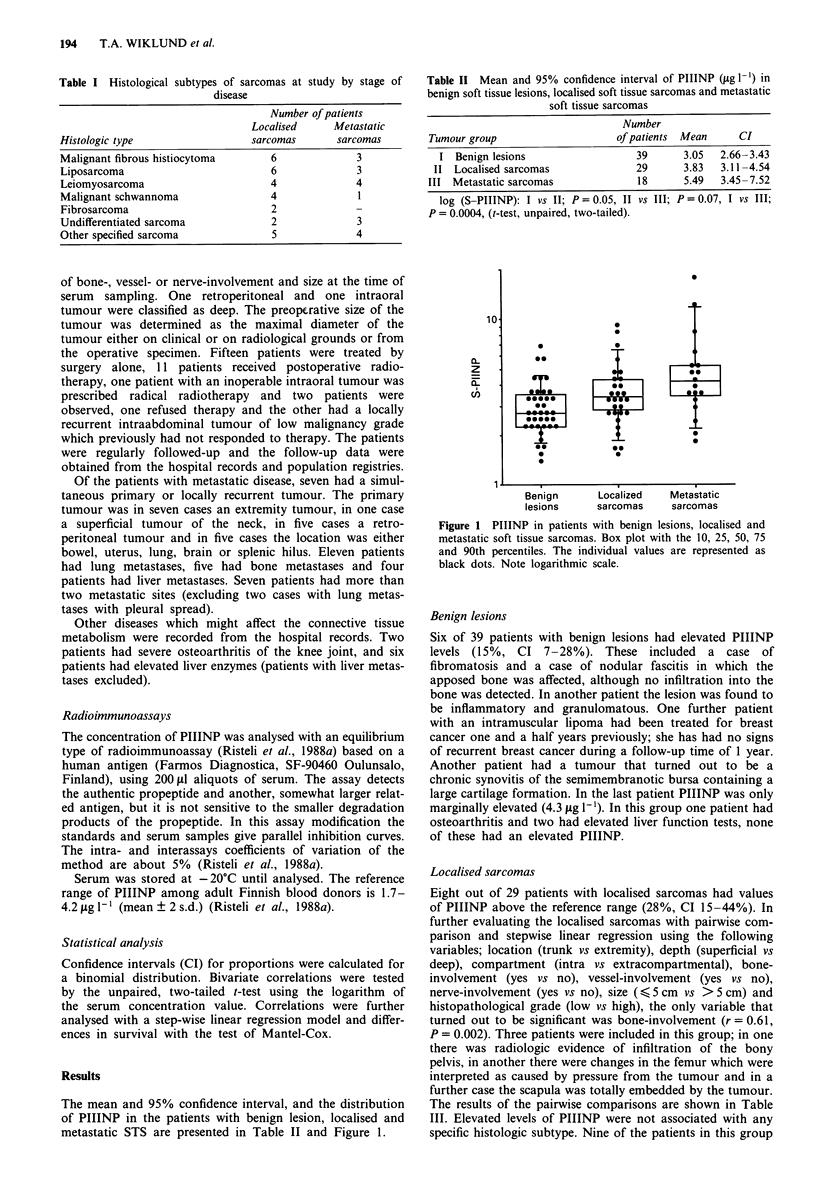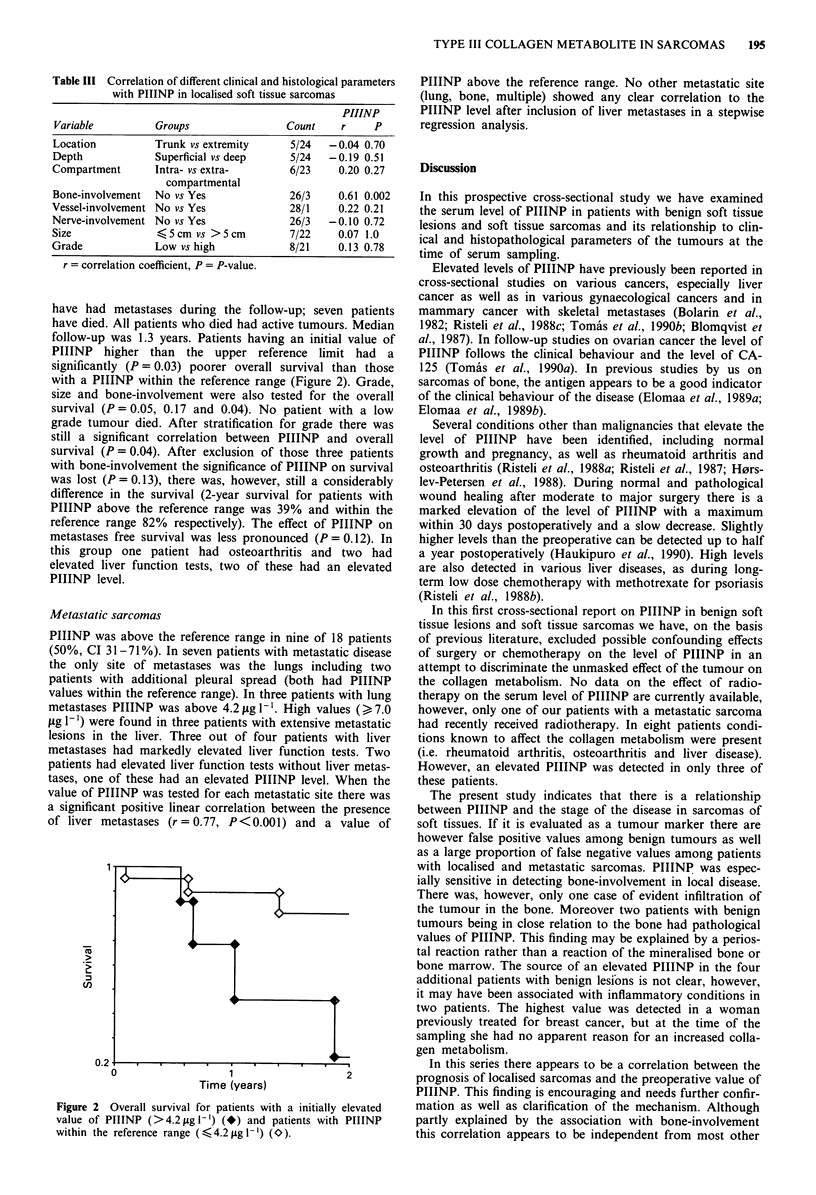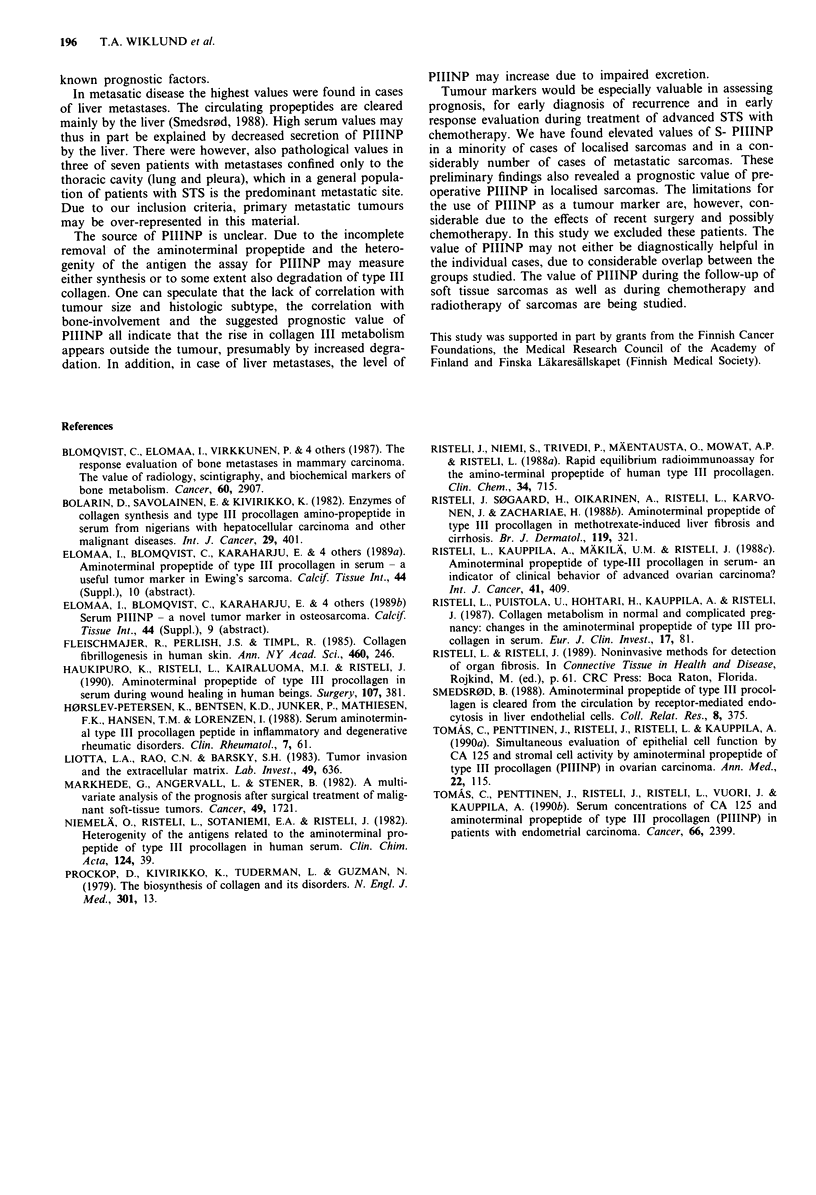# Type III collagen metabolism in soft tissue sarcomas.

**DOI:** 10.1038/bjc.1992.40

**Published:** 1992-02

**Authors:** T. A. Wiklund, I. Elomaa, C. P. Blomqvist, L. Risteli, J. Risteli

**Affiliations:** Department of Radiotherapy and Oncology, Helsinki University Central Hospital, Finland.

## Abstract

Sera of 85 patients with benign soft tissue lesions or sarcomas of soft tissues were investigated for a collagen metabolite, the aminoterminal propeptide of type III procollagen (PIIINP). Patients were divided into three groups: benign soft tissue lesions (n = 39), localised (n = 29) and metastatic (n = 18) soft tissue sarcomas (STS). Values of PIIINP above the reference range were found in 15%, 28% and 50% of the respective groups. The difference in the concentration of PIIINP was statistically significant between the benign lesions and the localised sarcomas; P = 0.05, and between the benign lesions and the metastatic sarcomas; P less than 0.001. In localised sarcomas there was a correlation between PIIINP and bone-involvement (r = 0.61, P = 0.002) and in metastatic disease between PIIINP and liver metastases (r = 0.77, P less than 0.001). In localised sarcomas the overall survival for patients with a value of PIIINP above the reference range was significantly poorer (P = 0.03) than for patients with values within the reference range, even after stratification for the histological malignancy grade of the tumours (P = 0.04).


Br. J. Cancer (1992), 65, 193-196 © Macmillan Press Ltd., 1992

### Type III collagen metabolism in soft tissue sarcomas

T.A. Wiklund[1], I. Elomaa[1], C.P. Blomqvist[1], L. Risteli[2] & J. Risteli[2]

[1]*Department of Radiotherapy and Oncology, Helsinki University Central Hospital, Haartmaninkatu 4, SF-00290 Helsinki;* [2]*Departments of Medical Biochemistry and Clinical Chemistry, University of Oulu, Kajaanintie 50, SF-90220 Oulu, Finland.*

**Summary** Sera of 85 patients with benign soft tissue lesions or sarcomas of soft tissues were investigated for a collagen metabolite, the aminoterminal propeptide of type III procollagen (PIIINP). Patients were divided into three groups: benign soft tissue lesions ($n = 39$), localised ($n = 29$) and metastatic ($n = 18$) soft tissue sarcomas (STS). Values of PIIINP above the reference range were found in 15%, 28% and 50% of the respective groups. The difference in the concentration of PIIINP was statistically significant between the benign lesions and the localised sarcomas; $P = 0.05$, and between the benign lesions and the metastatic sarcomas; $P < 0.001$. In localised sarcomas there was a correlation between PIIINP and bone-involvement ($r = 0.61$, $P = 0.002$) and in metastatic disease between PIIINP and liver metastases ($r = 0.77$, $P < 0.001$). In localised sarcomas the overall survival for patients with a value of PIIINP above the reference range was significantly poorer ($P = 0.03$) than for patients with values within the reference range, even after stratification for the histological malignancy grade of the tumours ($P = 0.04$).

In malignant disease connective tissue metabolism may be involved in different mechanisms. The tumour may degrade matrix components in association with invasion, and there may be an increased production of matrix components by the host and/or by the tumour (Liotta *et al.*, 1983). Several methods have been developed that can be used to detect increased extracellular matrix metabolism in the body by a simple study of blood samples (Risteli & Risteli, 1989). The assay for the aminoterminal propeptide of type III procollagen (PIIINP) has been commercially available and thus most often used. High levels of PIIINP have been found in patients with various cancers, particularly primary and secondary liver cancer (Bolarin *et al.*, 1982), but also in bone sarcomas (Elomaa *et al.*, 1989*a*; Elomaa *et al.*, 1989*b*).

Type III collagen is found mainly in tissues such as skin, arteries and the uterus, muscle, liver and lung, bone marrow and periosteum (Prockop *et al.*, 1979). During synthesis of interstitial fibre-forming collagens (types I, II, III and V), procollagens with additional propeptide extensions at both ends of the molecule are formed first. These propeptides are cleaved off before the formation of collagen fibrils. The aminoterminal propeptides of type III procollagen are, however, not constantly cleaved off. Such type III collagen molecules which have retained the aminoterminal propeptides, called type III pN collagen, are usually found on the surface of collagen fibres (Fleischmajer *et al.*, 1985).

The propeptide is mainly eliminated by the liver endothelial cell (Smedsrød, 1988). Because its half-life in serum is only a few minutes, its presence indicates active collagen synthesis and turnover (Smedsrød, 1988). Quantification of the PIIINP antigen has been achieved by specific radioimmunoassays (Risteli *et al.*, 1988*a*). The antigen in serum is, however, heterogeneous, due to forms larger and smaller than the authentic propeptide (Niemelä *et al.*, 1982). The interference caused by the small degradation products of the propeptide has been successfully eliminated in the assay modification used in this study (Risteli *et al.*, 1988*a*). It is not known, however, whether the large PIIINP antigens are aggregates containing the authentic propeptide, or degradation products of tissue type III pN collagen (Niemelä *et al.*, 1982).

The aim of the present study was to elaborate on whether there are differences in the serum concentration of PIIINP in patients with benign soft tissue lesions and soft tissue sarcomas (STS) of different stages, and to study whether the propeptide level could be correlated with any clinical findings or used as a prognostic marker.

### Materials and methods

### Patients

The material studied consists of all the patients attending Helsinki University Central Hospital for a verified or suspected non-osseous sarcoma between May 1988 and October 1990. Samples from patients with benign lesions and sarcomas without evidence of metastases at time of sampling were taken preoperatively and from patients with metastatic sarcomas before surgery was performed or chemotherapy started. Excluded were patients under the age of 16 years, patients operated on within 1 year before sampling (with the exception of patients having minor incision biopsies), and patients who had received chemotherapy within 1 year before sampling.

Eighty-six samples from 85 patients met our inclusion criteria. These were divided into three groups; patients with benign soft tissue lesions of the trunk and the extremities ($n = 39$), patients with sarcomas without evidence of metastases at the time of sampling ($n = 29$) and patients with metastases present at the time of sampling ($n = 18$). Seven localised and 40 metastatic sarcomas were excluded because of recent surgery or chemotherapy. In the analysis local recurrences were regarded as new primaries. In each group, however, each patient was included only once. All tumours were histologically confirmed with the exception of a few benign lesions whose benign nature were judged on clinical or radiological grounds. Tumours were histologically graded using a four-graded scale (Markhede *et al.*, 1982). In the analysis high grade tumours were those of malignancy grade three and four, and low grade tumours those of grade one and two.

Fourteen of the benign lesions were lipomas, five neurilemomas, three elastofibromas and two desmoid tumours, hemangiomas and myositis ossificans each. Eleven were miscellaneous soft tissue lesions or tumours, one of each.

The histological subtypes of the sarcomas are presented in Table I. Twenty-six of the localised sarcomas were extremity tumours, two were in the head and neck region (one superficial and one intraoral) and one was retroperitoneal. Five were locally recurrent tumours. Localised sarcomas of the trunk and the extremities were further classified as for tumour location and depth, compartmental status, presence

Correspondence: T. Wiklund, Department of Radiotherapy and Oncology, Helsinki University Central Hospital, Haartmaninkatu 4, SF-00290 Helsinki, Finland.
Received 17 July 1991; and in revised form 16 October 1991.

**Table I** Histological subtypes of sarcomas at study by stage of disease

| | *Number of patients* | |
|---|---|---|
| *Histologic type* | *Localised sarcomas* | *Metastatic sarcomas* |
| Malignant fibrous histiocytoma | 6 | 3 |
| Liposarcoma | 6 | 3 |
| Leiomyosarcoma | 4 | 4 |
| Malignant schwannoma | 4 | 1 |
| Fibrosarcoma | 2 | – |
| Undifferentiated sarcoma | 2 | 3 |
| Other specified sarcoma | 5 | 4 |

of bone-, vessel- or nerve-involvement and size at the time of serum sampling. One retroperitoneal and one intraoral tumour were classified as deep. The preoperative size of the tumour was determined as the maximal diameter of the tumour either on clinical or on radiological grounds or from the operative specimen. Fifteen patients were treated by surgery alone, 11 patients received postoperative radiotherapy, one patient with an inoperable intraoral tumour was prescribed radical radiotherapy and two patients were observed, one refused therapy and the other had a locally recurrent intraabdominal tumour of low malignancy grade which previously had not responded to therapy. The patients were regularly followed-up and the follow-up data were obtained from the hospital records and population registries.

Of the patients with metastatic disease, seven had a simultaneous primary or locally recurrent tumour. The primary tumour was in seven cases an extremity tumour, in one case a superficial tumour of the neck, in five cases a retroperitoneal tumour and in five cases the location was either bowel, uterus, lung, brain or splenic hilus. Eleven patients had lung metastases, five had bone metastases and four patients had liver metastases. Seven patients had more than two metastatic sites (excluding two cases with lung metastases with pleural spread).

Other diseases which might affect the connective tissue metabolism were recorded from the hospital records. Two patients had severe osteoarthritis of the knee joint, and six patients had elevated liver enzymes (patients with liver metastases excluded).

### *Radioimmunoassays*

The concentration of PIIINP was analysed with an equilibrium type of radioimmunoassay (Risteli *et al.*, 1988*a*) based on a human antigen (Farmos Diagnostica, SF-90460 Oulunsalo, Finland), using 200 µl aliquots of serum. The assay detects the authentic propeptide and another, somewhat larger related antigen, but it is not sensitive to the smaller degradation products of the propeptide. In this assay modification the standards and serum samples give parallel inhibition curves. The intra- and interassays coefficients of variation of the method are about 5% (Risteli *et al.*, 1988*a*).

Serum was stored at $-20$°C until analysed. The reference range of PIIINP among adult Finnish blood donors is 1.7–4.2 $\mu g\ l^{-1}$ (mean $\pm$ 2 s.d.) (Risteli *et al.*, 1988*a*).

### *Statistical analysis*

Confidence intervals (CI) for proportions were calculated for a binomial distribution. Bivariate correlations were tested by the unpaired, two-tailed *t*-test using the logarithm of the serum concentration value. Correlations were further analysed with a step-wise linear regression model and differences in survival with the test of Mantel-Cox.

### Results

The mean and 95% confidence interval, and the distribution of PIIINP in the patients with benign lesion, localised and metastatic STS are presented in Table II and Figure 1.

**Table II** Mean and 95% confidence interval of PIIINP ($\mu g\ l^{-1}$) in benign soft tissue lesions, localised soft tissue sarcomas and metastatic soft tissue sarcomas

| *Tumour group* | *Number of patients* | *Mean* | *CI* |
|---|---|---|---|
| I Benign lesions | 39 | 3.05 | 2.66–3.43 |
| II Localised sarcomas | 29 | 3.83 | 3.11–4.54 |
| III Metastatic sarcomas | 18 | 5.49 | 3.45–7.52 |

log (S-PIIINP): I *vs* II; $P = 0.05$, II *vs* III; $P = 0.07$, I *vs* III; $P = 0.0004$, (*t*-test, unpaired, two-tailed).

**Figure 1** PIIINP in patients with benign lesions, localised and metastatic soft tissue sarcomas. Box plot with the 10, 25, 50, 75 and 90th percentiles. The individual values are represented as black dots. Note logarithmic scale.

### *Benign lesions*

Six of 39 patients with benign lesions had elevated PIIINP levels (15%, CI 7–28%). These included a case of fibromatosis and a case of nodular fascitis in which the apposed bone was affected, although no infiltration into the bone was detected. In another patient the lesion was found to be inflammatory and granulomatous. One further patient with an intramuscular lipoma had been treated for breast cancer one and a half years previously; she has had no signs of recurrent breast cancer during a follow-up time of 1 year. Another patient had a tumour that turned out to be a chronic synovitis of the semimembranotic bursa containing a large cartilage formation. In the last patient PIIINP was only marginally elevated (4.3 $\mu g\ l^{-1}$). In this group one patient had osteoarthritis and two had elevated liver function tests, none of these had an elevated PIIINP.

### *Localised sarcomas*

Eight out of 29 patients with localised sarcomas had values of PIIINP above the reference range (28%, CI 15–44%). In further evaluating the localised sarcomas with pairwise comparison and stepwise linear regression using the following variables; location (trunk *vs* extremity), depth (superficial *vs* deep), compartment (intra *vs* extracompartmental), bone-involvement (yes *vs* no), vessel-involvement (yes *vs* no), nerve-involvement (yes *vs* no), size ($\leqslant$5 cm *vs* $>$5 cm) and histopathological grade (low *vs* high), the only variable that turned out to be significant was bone-involvement ($r = 0.61$, $P = 0.002$). Three patients were included in this group; in one there was radiologic evidence of infiltration of the bony pelvis, in another there were changes in the femur which were interpreted as caused by pressure from the tumour and in a further case the scapula was totally embedded by the tumour. The results of the pairwise comparisons are shown in Table III. Elevated levels of PIIINP were not associated with any specific histologic subtype. Nine of the patients in this group

**Table III** Correlation of different clinical and histological parameters with PIIINP in localised soft tissue sarcomas

| *Variable* | *Groups* | *Count* | *PIIINP* *r* | *P* |
|---|---|---|---|---|
| Location | Trunk *vs* extremity | 5/24 | −0.04 | 0.70 |
| Depth | Superficial *vs* deep | 5/24 | −0.19 | 0.51 |
| Compartment | Intra- *vs* extra-compartmental | 6/23 | 0.20 | 0.27 |
| Bone-involvement | No *vs* Yes | 26/3 | 0.61 | 0.002 |
| Vessel-involvement | No *vs* Yes | 28/1 | 0.22 | 0.21 |
| Nerve-involvement | No *vs* Yes | 26/3 | −0.10 | 0.72 |
| Size | ⩽5 cm *vs* >5 cm | 7/22 | 0.07 | 1.0 |
| Grade | Low *vs* high | 8/21 | 0.13 | 0.78 |

$r$ = correlation coefficient, $P$ = $P$-value.

have had metastases during the follow-up; seven patients have died. All patients who died had active tumours. Median follow-up was 1.3 years. Patients having an initial value of PIIINP higher than the upper reference limit had a significantly ($P = 0.03$) poorer overall survival than those with a PIIINP within the reference range (Figure 2). Grade, size and bone-involvement were also tested for the overall survival ($P = 0.05$, 0.17 and 0.04). No patient with a low grade tumour died. After stratification for grade there was still a significant correlation between PIIINP and overall survival ($P = 0.04$). After exclusion of those three patients with bone-involvement the significance of PIIINP on survival was lost ($P = 0.13$), there was, however, still a considerably difference in the survival (2-year survival for patients with PIIINP above the reference range was 39% and within the reference range 82% respectively). The effect of PIIINP on metastases free survival was less pronounced ($P = 0.12$). In this group one patient had osteoarthritis and two had elevated liver function tests, two of these had an elevated PIIINP level.

*Metastatic sarcomas*

PIIINP was above the reference range in nine of 18 patients (50%, CI 31–71%). In seven patients with metastatic disease the only site of metastases was the lungs including two patients with additional pleural spread (both had PIIINP values within the reference range). In three patients with lung metastases PIIINP was above 4.2 $\mu g\, l^{-1}$. High values ($\geqslant 7.0$ $\mu g\, l^{-1}$) were found in three patients with extensive metastatic lesions in the liver. Three out of four patients with liver metastases had markedly elevated liver function tests. Two patients had elevated liver function tests without liver metastases, one of these had an elevated PIIINP level. When the value of PIIINP was tested for each metastatic site there was a significant positive linear correlation between the presence of liver metastases ($r = 0.77$, $P < 0.001$) and a value of

**Figure 2** Overall survival for patients with a initially elevated value of PIIINP (>4.2 $\mu g\, l^{-1}$) (◆) and patients with PIIINP within the reference range (⩽4.2 $\mu g\, l^{-1}$) (◇).

PIIINP above the reference range. No other metastatic site (lung, bone, multiple) showed any clear correlation to the PIIINP level after inclusion of liver metastases in a stepwise regression analysis.

### Discussion

In this prospective cross-sectional study we have examined the serum level of PIIINP in patients with benign soft tissue lesions and soft tissue sarcomas and its relationship to clinical and histopathological parameters of the tumours at the time of serum sampling.

Elevated levels of PIIINP have previously been reported in cross-sectional studies on various cancers, especially liver cancer as well as in various gynaecological cancers and in mammary cancer with skeletal metastases (Bolarin *et al.*, 1982; Risteli *et al.*, 1988*c*; Tomás *et al.*, 1990*b*; Blomqvist *et al.*, 1987). In follow-up studies on ovarian cancer the level of PIIINP follows the clinical behaviour and the level of CA-125 (Tomás *et al.*, 1990*a*). In previous studies by us on sarcomas of bone, the antigen appears to be a good indicator of the clinical behaviour of the disease (Elomaa *et al.*, 1989*a*; Elomaa *et al.*, 1989*b*).

Several conditions other than malignancies that elevate the level of PIIINP have been identified, including normal growth and pregnancy, as well as rheumatoid arthritis and osteoarthritis (Risteli *et al.*, 1988*a*; Risteli *et al.*, 1987; Hørslev-Petersen *et al.*, 1988). During normal and pathological wound healing after moderate to major surgery there is a marked elevation of the level of PIIINP with a maximum within 30 days postoperatively and a slow decrease. Slightly higher levels than the preoperative can be detected up to half a year postoperatively (Haukipuro *et al.*, 1990). High levels are also detected in various liver diseases, as during long-term low dose chemotherapy with methotrexate for psoriasis (Risteli *et al.*, 1988*b*).

In this first cross-sectional report on PIIINP in benign soft tissue lesions and soft tissue sarcomas we have, on the basis of previous literature, excluded possible confounding effects of surgery or chemotherapy on the level of PIIINP in an attempt to discriminate the unmasked effect of the tumour on the collagen metabolism. No data on the effect of radiotherapy on the serum level of PIIINP are currently available, however, only one of our patients with a metastatic sarcoma had recently received radiotherapy. In eight patients conditions known to affect the collagen metabolism were present (i.e. rheumatoid arthritis, osteoarthritis and liver disease). However, an elevated PIIINP was detected in only three of these patients.

The present study indicates that there is a relationship between PIIINP and the stage of the disease in sarcomas of soft tissues. If it is evaluated as a tumour marker there are however false positive values among benign tumours as well as a large proportion of false negative values among patients with localised and metastatic sarcomas. PIIINP was especially sensitive in detecting bone-involvement in local disease. There was, however, only one case of evident infiltration of the tumour in the bone. Moreover two patients with benign tumours being in close relation to the bone had pathological values of PIIINP. This finding may be explained by a periostal reaction rather than a reaction of the mineralised bone or bone marrow. The source of an elevated PIIINP in the four additional patients with benign lesions is not clear, however, it may have been associated with inflammatory conditions in two patients. The highest value was detected in a woman previously treated for breast cancer, but at the time of the sampling she had no apparent reason for an increased collagen metabolism.

In this series there appears to be a correlation between the prognosis of localised sarcomas and the preoperative value of PIIINP. This finding is encouraging and needs further confirmation as well as clarification of the mechanism. Although partly explained by the association with bone-involvement this correlation appears to be independent from most other known prognostic factors.

In metasatic disease the highest values were found in cases of liver metastases. The circulating propeptides are cleared mainly by the liver (Smedsrød, 1988). High serum values may thus in part be explained by decreased secretion of PIIINP by the liver. There were however, also pathological values in three of seven patients with metastases confined only to the thoracic cavity (lung and pleura), which in a general population of patients with STS is the predominant metastatic site. Due to our inclusion criteria, primary metastatic tumours may be over-represented in this material.

The source of PIIINP is unclear. Due to the incomplete removal of the aminoterminal propeptide and the heterogenity of the antigen the assay for PIIINP may measure either synthesis or to some extent also degradation of type III collagen. One can speculate that the lack of correlation with tumour size and histologic subtype, the correlation with bone-involvement and the suggested prognostic value of PIIINP all indicate that the rise in collagen III metabolism appears outside the tumour, presumably by increased degradation. In addition, in case of liver metastases, the level of PIIINP may increase due to impaired excretion.

Tumour markers would be especially valuable in assessing prognosis, for early diagnosis of recurrence and in early response evaluation during treatment of advanced STS with chemotherapy. We have found elevated values of S- PIIINP in a minority of cases of localised sarcomas and in a considerably number of cases of metastatic sarcomas. These preliminary findings also revealed a prognostic value of preoperative PIIINP in localised sarcomas. The limitations for the use of PIIINP as a tumour marker are, however, considerable due to the effects of recent surgery and possibly chemotherapy. In this study we excluded these patients. The value of PIIINP may not either be diagnostically helpful in the individual cases, due to considerable overlap between the groups studied. The value of PIIINP during the follow-up of soft tissue sarcomas as well as during chemotherapy and radiotherapy of sarcomas are being studied.

This study was supported in part by grants from the Finnish Cancer Foundations, the Medical Research Council of the Academy of Finland and Finska Läkaresällskapet (Finnish Medical Society).

### References

BLOMQVIST, C., ELOMAA, I., VIRKKUNEN, P. & 4 others (1987). The response evaluation of bone metastases in mammary carcinoma. The value of radiology, scintigraphy, and biochemical markers of bone metabolism. *Cancer*, **60**, 2907.

BOLARIN, D., SAVOLAINEN, E. & KIVIRIKKO, K. (1982). Enzymes of collagen synthesis and type III procollagen amino-propeptide in serum from nigerians with hepatocellular carcinoma and other malignant diseases. *Int. J. Cancer*, **29**, 401.

ELOMAA, I., BLOMQVIST, C., KARAHARJU, E. & 4 others (1989*a*). Aminoterminal propeptide of type III procollagen in serum – a useful tumor marker in Ewing's sarcoma. *Calcif. Tissue Int.*, **44** (Suppl.), 10 (abstract).

ELOMAA, I., BLOMQVIST, C., KARAHARJU, E. & 4 others (1989*b*) Serum PIIINP – a novel tumor marker in osteosarcoma. *Calcif. Tissue Int.*, **44** (Suppl.), 9 (abstract).

FLEISCHMAJER, R., PERLISH, J.S. & TIMPL, R. (1985). Collagen fibrillogenesis in human skin. *Ann. NY Acad. Sci.*, **460**, 246.

HAUKIPURO, K., RISTELI, L., KAIRALUOMA, M.I. & RISTELI, J. (1990). Aminoterminal propeptide of type III procollagen in serum during wound healing in human beings. *Surgery*, **107**, 381.

HØRSLEV-PETERSEN, K., BENTSEN, K.D., JUNKER, P., MATHIESEN, F.K., HANSEN, T.M. & LORENZEN, I. (1988). Serum aminoterminal type III procollagen peptide in inflammatory and degenerative rheumatic disorders. *Clin. Rheumatol.*, **7**, 61.

LIOTTA, L.A., RAO, C.N. & BARSKY, S.H. (1983). Tumor invasion and the extracellular matrix. *Lab. Invest.*, **49**, 636.

MARKHEDE, G., ANGERVALL, L. & STENER, B. (1982). A multivariate analysis of the prognosis after surgical treatment of malignant soft-tissue tumors. *Cancer*, **49**, 1721.

NIEMELÄ, O., RISTELI, L., SOTANIEMI, E.A. & RISTELI, J. (1982). Heterogenity of the antigens related to the aminoterminal propeptide of type III procollagen in human serum. *Clin. Chim. Acta*, **124**, 39.

PROCKOP, D., KIVIRIKKO, K., TUDERMAN, L. & GUZMAN, N. (1979). The biosynthesis of collagen and its disorders. *N. Engl. J. Med.*, **301**, 13.

RISTELI, J., NIEMI, S., TRIVEDI, P., MÄENTAUSTA, O., MOWAT, A.P. & RISTELI, L. (1988*a*). Rapid equilibrium radioimmunoassay for the amino-terminal propeptide of human type III procollagen. *Clin. Chem.*, **34**, 715.

RISTELI, J. SØGAARD, H., OIKARINEN, A., RISTELI, L., KARVONEN, J. & ZACHARIAE, H. (1988*b*). Aminoterminal propeptide of type III procollagen in methotrexate-induced liver fibrosis and cirrhosis. *Br. J. Dermatol.*, **119**, 321.

RISTELI, L., KAUPPILA, A., MÄKILÄ, U.M. & RISTELI, J. (1988*c*). Aminoterminal propeptide of type-III procollagen in serum- an indicator of clinical behavior of advanced ovarian carcinoma? *Int. J. Cancer*, **41**, 409.

RISTELI, L., PUISTOLA, U., HOHTARI, H., KAUPPILA, A. & RISTELI, J. (1987). Collagen metabolism in normal and complicated pregnancy: changes in the aminoterminal propeptide of type III procollagen in serum. *Eur. J. Clin. Invest.*, **17**, 81.

RISTELI, L. & RISTELI, J. (1989). Noninvasive methods for detection of organ fibrosis. In *Connective Tissue in Health and Disease*, Rojkind, M. (ed.), p. 61. CRC Press: Boca Raton, Florida.

SMEDSRØD, B. (1988). Aminoterminal propeptide of type III procollagen is cleared from the circulation by receptor-mediated endocytosis in liver endothelial cells. *Coll. Relat. Res.*, **8**, 375.

TOMÁS, C., PENTTINEN, J., RISTELI, J., RISTELI, L. & KAUPPILA, A. (1990*a*). Simultaneous evaluation of epithelial cell function by CA 125 and stromal cell activity by aminoterminal propeptide of type III procollagen (PIIINP) in ovarian carcinoma. *Ann. Med.*, **22**, 115.

TOMÁS, C., PENTTINEN, J., RISTELI, J., RISTELI, L., VUORI, J. & KAUPPILA, A. (1990*b*). Serum concentrations of CA 125 and aminoterminal propeptide of type III procollagen (PIIINP) in patients with endometrial carcinoma. *Cancer*, **66**, 2399.